# Perspectives of basic wheelchair users on improving their access to wheelchair services in Kenya and Philippines: a qualitative study

**DOI:** 10.1186/s12914-017-0130-6

**Published:** 2017-08-17

**Authors:** Emma Williams, Elizabeth Hurwitz, Immaculate Obaga, Brenda Onguti, Adovich Rivera, Tyrone Reden L. Sy, R. Lee Kirby, Jamie Noon, Deepti Tanuku, Anthony Gichangi, Eva Bazant

**Affiliations:** 1 Jhpiego, 1615 Thames St, Baltimore, MD 21231-3492 USA; 2 Jhpiego, 14 Riverside (Off Riverside Drive) Arlington Block, 2nd Floor, P.O Box 66119-00800, Nairobi, Kenya; 3 Institute of Health Policy and Development Studies, University of Philippines, Room 105, National Institutes of Health Building, 623 Pedro Gil St., Ermita, 1000 Manila, Philippines; 4 Dalhousie University, c/o Room 206, Nova Scotia Rehabilitation Centre Site, 1341 Summer Street, Halifax, NS B3H 4K4 Canada; 5 Noon Design, Box 208, Cerrillos, NM 87010 USA

**Keywords:** Wheelchairs, Health services, Africa, Asia, Qualitative, Kenya, Philippines

## Abstract

**Background:**

The United Nations has called for countries to improve access to mobility devices when needed. The World Health Organization has published guidelines on the provision of manual wheelchairs in less-resourced settings. Yet little is known about the extent to which appropriate wheelchairs are available and provided according to international guidelines. This study’s purpose was to describe wheelchair users’ experiences receiving services and acquiring wheelchair skills in urban and peri-urban areas of Kenya and the Philippines.

**Methods:**

Local researchers in Nairobi and Manila interviewed 48 adult basic wheelchair users, with even distribution of those who had and had not received wheelchair services along with their wheelchair. Recordings were transcribed in the local language and translated into English. The study team coded transcripts for predetermined and emergent themes, using Atlas-ti software. A qualitative content analysis approach was taken with the WHO service delivery process as an organizing framework.

**Results:**

Wheelchair users frequently described past experiences with ill-fitting wheelchairs and little formal training to use wheelchairs effectively. Through exposure to multiple wheelchairs and self-advocacy, they learned to select wheelchairs suitable for their needs. Maintenance and repair services were often in short supply. Participants attributed shorter duration of wheelchair use to lack of repair. Peer support networks emerged as an important source of knowledge, resources and emotional support. Most participants acknowledged that they received wheelchairs that would have been difficult or impossible for them to pay for, and despite challenges, they were grateful to have some means of mobility. Four themes emerged as critical for understanding the implementation of wheelchair services: barriers in the physical environment, the need for having multiple chairs to improve access, perceived social stigma, and the importance of peer support.

**Conclusions:**

Interventions are needed to provide wheelchairs services efficiently, at scale, in an environment facilitating physical access and peer support, and reduced social stigma.

**Trial registration:**

Not applicable since this was a descriptive study.

**Electronic supplementary material:**

The online version of this article (doi:10.1186/s12914-017-0130-6) contains supplementary material, which is available to authorized users.

## Background

The United Nations has affirmed the right for persons with disabilities to have access to affordable mobility devices, social inclusion, and community participation [1]. Disability is strongly associated with poverty, and most people in less-resourced settings who need wheelchairs are unable to buy their own wheelchairs [2]. Approximately 15% of the world’s population have some type of disability, and 1% of the population globally need wheelchairs for increased mobility, although precise estimates for less-resourced settings are unavailable [2]. More than 300,000 wheelchairs are donated annually to low and middle income countries by international donors and charitable organizations; this includes an estimated 900 per year in Kenya and 4000 per year in the Philippines [3]. Often this process is outside the formal health care system [4].

When a wheelchair is provided to a person, other services are needed to improve the chance that the wheelchair will enable improved quality of life and social functioning. In 2008, the World Health Organization (WHO) published guidelines on the provision of manual wheelchairs in less-resourced settings, as well as training packages for service providers [5]. These guidelines included eight steps for service delivery, as presented in Table 1.

**Table 1:** Wheelchair Service-Delivery Steps. Reprinted with permission from *Guidelines on the provision of manual wheelchairs in less resourced settings* ^*a*^

Step		Summary
1	Referral and appointment	The system of referral will depend on existing services in the country. Users may self-refer or be referred through networks made up of governmental or nongovernmental health and rehabilitation workers or volunteers working at community, district or national level. Some services may need to actively identify potential users if they are not already receiving any social or health care services or participating in school work on community activities.
2	Assessment	Each user requires an individual assessment, taking into account lifestyle, vocation, home environment and physical condition.
3	Prescription (selection)	Using the information gained from the assessment, a wheelchair prescription is developed together with the user, family member, or caregiver. The prescription details the selected wheelchair type, size, special features or modifications. Also detailed is the training the user needs to effectively use and maintain the wheelchair.
4	Funding and ordering	A funding source is identified and the wheelchair is ordered from the stock held by the service or from the provider.
5	Product preparation	Trained personnel prepare the wheelchair for the initial fitting. Depending on the product or service facilities, this may include assembly and possible modification, of products supplied by manufacturers or production of products in the service workshop.
6	Fitting	The user tries the wheelchair. Final adjustments are made to ensure the wheelchair is correctly assembled and set up. If modifications or postural support components are required, additional fittings may be necessary.
7	User training	The user and caregivers are instructed on how to safely and effectively use and maintain the wheelchair.
8	Follow-up, maintenance and repairs	Follow-up appointments are an opportunity to check wheelchair fit and provide further training and support. The timing depends on the needs of the user and the other services that are available to them. The service may also offer maintenance and repairs for technical problems that cannot be easily solved in the community. It is appropriate to carry out follow-up activities at the community level as much as possible. If the wheelchair is found to be no longer appropriate, a new wheelchair needs to be supplied starting again from step 1.

^a^Available online at: http://www.who.int/disabilities/publications/technology/wheelchairguidelines/en

A few studies have explored to what extent wheelchair service guidelines are implemented in low and middle-income countries [6]. One longitudinal study from Indonesia found that adults and children who received wheelchair services according to WHO guidelines had improved outcomes compared to wait listed controls [7]. One cross-sectional survey in Bangladesh of wheelchair users and -individuals with hearing impairment found some aspects of services (being asked about their needs, being measured and receiving training) were associated with some outcomes, including wheelchair satisfaction, participation and quality of life [8]. A review of wheelchair service provision in Afghanistan, India, Kosovo and Zimbabwe found that the needs and service distribution models vary by setting [4]. A study from India assessed 167 recipients of wheelchairs from charitable organizations and found that only 18% were still using the wheelchairs that they were given [9]. In a study in Beijing, China, of wheelchair users and persons with other disabilities, survey-based needs assessment related to rehabilitation and a quantitative assessment of barriers to rehabilitation services [10].

Fewer studies have employed qualitative methods to capture wheelchair users’ experiences in their own words and specifically examine how wheelchair recipients experience wheelchair provision and related services. In a small mixed methods study from Zimbabwe using two focus group discussions and two case studies [11] found that while wheelchair users appreciated receiving wheelchairs, they often faced challenges related to poor fit, lack of training in proper maneuvering of the wheelchair, and frequent needs to repair tires and other parts.

Thus, descriptive data is largely absent regarding how wheelchair services have been implemented in low and middle income countries and the extent to which wheelchair users perceive these services to be responsive to their needs [12]. The purpose of this qualitative study was to describe the services that urban and peri-urban wheelchair users received in Kenya and the Philippines and the perceived value of receiving a wheelchair with services or receiving a wheelchair without accompanying services.

## Methods

### Study setting

The study team selected Kenya and the Philippines as the study sites in collaboration with the donor, expert advisors, and organizations that provide wheelchairs in Africa and Asia. in order to (a) include one country each from Asia and Africa that are broadly representative of other countries in East Africa and the Asia Pacific, (b) include countries with large volume of wheelchair provision both with and without accompanying services, (c) obtain lists of wheelchair recipients. The study sites were further limited to urban and peri-urban areas, because in rural areas it would have been difficult to recruit sufficient numbers of wheelchair users within the time and budget restrictions.

Data collection took place in and around Nairobi from December 2014 through May 2015 and in the greater Manila area from February to May 2015. Participants in Kenya were mainly recruited from 3 urban and peri-urban counties near the capital city - Nairobi, Machakos and Kiambu - which account for 15% of the Kenyan population according to the 2009 Kenya population and housing census report [13]. (One participant was from Nakuru and two were from Mombasa.) The 2008 Kenya National Survey for persons with disability reported an overall disability rate of 4.6%, of which 1.6% of the Kenyan population has some physical impairment [10]. Although Kenya’s Persons with Disability Act of 2003 provides a legislative framework for access to services and inclusion of persons with disabilities in all facets of life, a 2014 status report on the implementation of the rights of persons with disabilities pointed out many challenges to persons with disabilities, including discrimination and stigma, and physical inaccessibility of buildings and transportation services [11].

As the capital region of the Philippines, the Metro Manila area is a highly urbanized district with a population of almost 12 million. An estimated 1.6% of the population are people living with disabilities, according to 2010 census data [14]. In the Philippines, laws exist to protect the rights of persons with disabilities - such as employment and educational equality - and provide discounts to some basic goods and services [15]. The social health insurance program does not cover wheelchair provision and services; however, some government social welfare offices offer free wheelchairs that have been donated by charitable organizations.

### Ethical issues

The study was approved by the research ethics boards of Johns Hopkins University Bloomberg School of Public Health in Baltimore, Maryland, United States (#5839), the Kenya Medical Research Institute in Nairobi, Kenya (Non-SSC Determination #457) and the University of Philippines Manila (#2014-351-01).

All study participants provided informed consent; oral in Kenya and written in Philippines, based on local institutional review board preferences.

### Study design

The qualitative study sample was a subset of participants in a survey of 852 wheelchair users in Kenya and the Philippines conducted between December 2014 and May 2015. The survey methods and quantitative results have been published elsewhere [13, 15]. The rationale for employing qualitative data collection was to describe wheelchair service provision in wheelchair users’ own voices and to place this in the context of wheelchair users’ lives.

### Participants, recruitment and screening

In Kenya, the study team recruited potential participants from lists provided by (a) 10 wheelchair-providing organizations, such as faith-based organizations, nongovernmental organizations, community-based organizations, and government hospitals; (b) 11 disabled persons’ organizations; and (c) snowball sampling (referral from other study participants). Surveyors and field supervisors prescreened participants by phone and scheduled appointments at wheelchair users’ homes or other accessible locations. In the Philippines, the team recruited from lists provided by (a) five local government units (LGUs) within metropolitan Manila, which provide free wheelchairs to residents; (b) a charitable organization that provides free wheelchairs through LGUs, civic organizations and other organizations; (c) a nongovernmental organization where wheelchair users live and work; and (d) snowball sampling. Potential participants were screened over the phone, if possible, or contacted by visiting their homes.

### Inclusion and exclusion criteria

Participants were at least 18 years old and basic manual wheelchairs users, meaning that they required no postural support to remain in a seated position. Eligible respondents received their current wheelchairs at least six months and less than five years prior to data collection. In the Philippines only, in response to challenges in recruiting a large enough sample, toward the end of data collection, users who had received their wheelchair 10 years earlier were included to increase the participation of users of rugged wheelchairs, which might last longer and be delivered in conjunction with services. Study enrollment was preceded by screening to determine if potential participants had ever received wheelchair services; details are provided elsewhere [13, 15]. All qualitative interview participants had first completed the survey. One participant in Kenya completed the survey but refused to complete the in-depth interview. No interview participants refused in the Philippines.

### Qualitative interviews

A team of two experienced interviewers in Kenya and seven in the Philippines completed 24 in-depth interviews in each country, purposively selected to include even distribution based on sex, receipt of any services with their most recent wheelchair, age (a binary categorization of younger or older than 45 years) and geographical area*.* We stratified the sample this way to include diverse opinions. Because we had no reliable information about the age distribution or life expectancy of wheelchair users, age 45 was somewhat arbitrarily chosen as a cut-off for older users.

The interview guide included open-ended questions about experiences with wheelchairs, wheelchair services and contextual factors and was translated into Swahili and Filipino. (Additional file 1). Interviews took place in participants’ homes. Interviewers sought a location with auditory privacy, and no one else was present for the interview, except for one participant in the Philippines who was caring for his child during the interview. Interviewers used digital voice recorders during the interviews. Interviewers transcribed the audio files and then translation into English was done by the interviewers in Kenya and the Center for the Filipino Language at the University of the Philippines in Manila; in the Philippines, field interviewers checked the completed English translations against the Filipino transcriptions to identify translation errors.

#### Data analysis and interpretation

A qualitative content analysis approach was employed [16]. Data from the two countries were analyzed separately. Members of the research team coded the transcripts with Atlas-ti software, using a code list created based on WHO guidelines for service delivery. Coders added emergent codes based on multiple-person coding of a subset of transcripts, and more codes were added as the process continued. Strategies to improve consistency across coders included developing standard code definitions, double-coding a subset of transcripts, and holding regular discussions among coders. In addition to the coding, the first author also read the transcripts in their entirety several times. Using the computer-assisted Noticing-Collecting-Thinking approach of Friese [17], analysts queried the data to generate reports based on codes and respondent characteristics and identified patterns in the data. The first author wrote memos to synthesize the findings. The qualitative team was in frequent communication to discuss the findings. For this manuscript, participants were given a unique identifier starting with K for Kenya or P for Philippines and ending with a two-digit number that was different from the identifier used by the study team. This code is provided to enable to readers to understand whether the the participants were quoted more than once.

Data for steps 1-2 (referral and appointment, and assessment) and for steps 3-4 (prescription, and funding and ordering) were combined because in these settings they occurred simultaneously. Similarly, data. Findings related to step 5 are not presented because wheelchair users were rarely present during the product preparation stage. Four themes emerged as critical for understanding the implementation of wheelchair services: barriers in the physical environment, the need for having multiple chairs to improve access, perceived social stigma, and the importance of peer support.

In August 2015, representatives of the research team and stakeholders met for two days in each country to disseminate study findings; meeting attendees included more than 100 representatives of government agencies, nongovernmental organizations, disabled persons organizations, local universities, wheelchair manufacturers, and wheelchair professionals, and their responses to the findings were taken into account for this article and served as a form of member checking.

## Results

### Overview of respondents

In Kenya, 9 of qualitative interview participants were less than 35 years old, the most common reason for using a wheelchair was spinal cord injury (11), and 18 said they used their wheelchair for at least eight hours per day (Table 2). In the Philippines, four participants were less than 35 years old, had needed wheelchair was complications of polio (9), and 10 used their wheelchair for at least eight hours per day. Twelve participants in the Philippines and eight in Kenya were currently married or cohabiting. Most respondents had at least a secondary education. They were evenly split between men and women.

**Table 2:** Characteristics of qualitative interview participants and their current wheelchairs, in Kenya and Philippines, based on questionnaire data

	Kenya		Philippines	
Age	Number	Percentage	Number	Percentage
18-34	9	39.1	4	16.7
35-49	7	30.4	10	41.7
50+	7	30.4	10	41.7
Male gender	12	52.2	12	50
Highest education attained				
Primary	7	30.4	7	29.2
Secondary, Post-Sec, Vocational	9	39.1	8	33.3
College or University	7	30.4	9	37.5
Marital status				
Married or cohabiting	8	36.4	12	50
Never married or cohabiting	11	50.0	9	37.5
Divorced, separated, or widowed	3	13.6	3	12.5
Employment status				
Unemployed	6	26.1	8	33.3
Trading or selling	1	4.3	6	25.0
Student	4	17.4	1	4.2
Craftsman	3	13.0	3	12.5
Other	9	39.1	6	25
Condition that led to wheelchair use				
Spinal cord injury	11	47.8	5	20.8
Polio/postpolio	5	21.7	9	37.5
Congenital	4	17.4	2	8.3
Other	3	13	8	33.3
Source of current wheelchair				
Mission hospital	2	8.7		
Government	1	4.3	11	45.8
Charitable organization	11	47.8	5	20.8
Pharmacy or medical supply store	1	4.3	2	8.3
Friend or family	4	17.4	2	8.3
Other	4	26.1	4	16.7
Current wheelchair was free	21	91.3	17	70.8
Type of wheelchair				
Basic indoor chair	13	56.5	22	91.7
Rough terrain chair	10	43.4	2	8.3
Level of wheelchair use				
Not daily	1	4.3	8	33.3
1-7 h daily	4	17.4	6	25
8+ hours daily	18	78.3	10	41.7
Total^b^	23	100	24	100

^a^Includes one don’t know in Kenya; ^b^One missing in Kenya

Other characteristics were elicited from the interviews rather than the survey data. Study participants ranged from those with robust physical health and strength to those with complex morbidities, and their duration of wheelchair use varied from a few years to several decades. Some participants were able to use crutches or braces to walk, or even walk independently for short distances, while others were unable to walk independently and used no mobility devices other than wheelchairs. The sample included those had attended schools for children with disabilities and thus had always had a peer group that included wheelchair users, as well as people who had little contact with other wheelchair users and felt isolated by their mobility impairment. Only two in-depth interview participants in Kenya and three in the Philippines had received their first wheelchair during the past five years. Participants had used up to 12 wheelchairs in Kenya and four wheelchairs in Philippines during their lifetimes, with an average of about 5 in Kenya and 2.5 in the Philippines, but some respondents were uncertain about the total number of wheelchairs that they had owned.

Findings below are presented according to the service steps outlined in Table 1.

### Steps 1-2: Referral, appointment, and assessment

Because of the sampling approach, most participants had received their wheelchairs for free from either local government officials (in the Philippines only) or charitable organizations, and referral occurred through a combination of luck and social networks. Generally, wheelchairs were described as being readily available. One Kenyan man said:

*I don’t ask for anyone to bring me a wheelchair. . . . I have not stayed with a wheelchair for a long time. . . . I just stay for some time and after about two years somebody comes or an organization comes. For, like an example, some people come here in school and say that we have brought you some wheelchairs, and we need you to use them. So I move to the next one. [K22]*

Less commonly, healthcare workers referred respondents through the health care system, such as during a hospital stay. Others described buying their new or used wheelchairs either in a market or shop or directly from other wheelchair users. Those who received wheelchairs from charitable organizations sometimes described being measured or otherwise assessed prior to being presented with wheelchairs, as well as being asked to submit some identifying information; photographs, especially in Kenya, were required from potential recipients. In other cases, recipients were assessed at the same time the wheelchairs were given or they were assigned wheelchairs without any assessments being done. Occasionally, well-meaning individuals gifted participants with wheelchairs, as surprise gestures.

Generally, participants could not describe the steps taken to prepare the wheelchairs for their use, since they received wheelchairs that had already been assembled.

### Steps 3-4: Prescription or selection, funding and ordering

Although some participants described an active prescription and selection process, many said that few choices in type of wheelchair were available in their community or few choices were presented to them. Some of those who received their wheelchairs through donation said they did not expect to receive a choice in wheelchairs or provide input in selection, since they were free, particularly for newer wheelchair users. However, some described negotiating with wheelchair providers to try to get a different wheelchair. In the Philippines, some described miscommunication or misunderstanding regarding the types of wheelchairs they were expecting to receive and the ones that they received. One man from the Philippines said that he was expecting to receive a wheelchair built for rugged outdoor settings (called a Roughrider) but he said:

*What arrived was a medical wheelchair. Of course that was given, what, you'll still complain?Ah, it was already here. And it's big! So, they arrived all at the same time, many arrived. I overheard [the doctor who distributed the wheelchairs] saying that there's one more that's quite small. I said, "Doc, maybe it's possible for me to replace it with anything." [She said] "No. You're better there! Because it's big. You're big." [P14]*

He went on to say that he still felt the wheelchair was too big and that he had added a piece of plywood to the seat and made other modifications to try to make the chair more comfortable.

Some respondents described how the wheelchairs had been unsuitable, but they only realized this later after having a chance to use other wheelchairs. With experience, users described more actively selecting wheelchairs to suit their needs. Experienced users desired choice in wheelchair selection, and an opportunity to try prospective wheelchairs, more than they wanted recommendations from service providers. However, they recommended that new wheelchair users be given counseling and training in wheelchair selection.

Because the sampling strategy primarily recruited people who had received their wheelchairs from charitable organizations or through a government program for those who could not afford to buy wheelchairs (in the Philippines only), most participants had received free wheelchairs. Users who paid for all or part of their wheelchair were often more engaged in the selection process and expressed more satisfaction with the wheelchairs, compared to users whose wheelchairs had been given to them.

### Step 5: Product preparation

Little data was available about product preparation since participants generally received fully assembled wheelchairs. Some participants described modifying the footrests, armrests or cushions on their own wheelchairs. Some added pockets or bags for carrying their necessities. Changing the cushion was one of the most common modifications described, to improve comfort, to reduce the risk of pressure sores, and to adjust wheelchairs that were too large or too low to the ground. In the Philippines, some participants lived near a wheelchair manufacturing facility and could ask employees to help make major modifications to their wheelchairs with welding. One participant in the Philippines designed his own wheelchair and had it manufactured, and another improvised a wooden chair with wheels attached, because his previous wheelchair tipped over too easily. In Kenya, users often had multiple wheelchairs for showering and indoor and outdoor use.

### Step 6: Fitting

Fitting was described as a range of experiences, from none at all to both the measurement of the body and adjustment of the wheelchair. Many participants, particularly those with longer experiences using wheelchairs or those who had used wheelchairs as children, had past experience receiving wheelchairs that fit poorly. As with selection, some recipients who received donated wheelchairs were not in a position to request fitting. One Kenyan man said:

*They think a wheelchair is a wheelchair. No, a wheelchair is supposed to fit you, but how do you get to the one fitting you if it is a donation? You know you want to save every cent when you are in this condition ‘coz there are other expenses definitely. [K5]*

Often participants were unaware of the value of fitting when they received their first wheelchairs and developed preferences over time by wearing out and replacing wheelchairs. A student from the Philippines, who had paraplegia resulting from a spinal cord injury and lived in a dormitory with other persons with disabilities, described two different experiences with fitting. First, he received a hospital-type wheelchair that was too large and caused “bed sores,” so he started using crutches instead. When the crutches became frustrating, he bought a used wheelchair from a fellow wheelchair user, after briefly sitting in the wheelchair and being advised by other wheelchairs users that it seemed like a good fit for him.

### Step 7: User training

Generally, study participants had received little formal training in how to maneuver their wheelchairs or solve problems related to wheelchair use, such as adapting to public bathrooms. Exceptions were those who stayed in a hospital for a long time and received physical therapy, or those who received some other kind of institutional care, such as in a residential school for children with disabilities. Instead, most described teaching themselves to use the wheelchair through practice, which one Kenyan man called “by feel, learning through experience, the hard way.” [K3] They might learn a few skills through brief training that was offered along with each new wheelchair. One Kenyan man received written booklets that helped him understand how to better maneuver his wheelchair. Some participants thought that new wheelchair recipients could benefit from training but that they themselves had figured out what they needed to know. Yet, even these experienced users had problems with some types of wheelchairs.

Frequently people spoke about light wheelchairs that tipped easily or rolled too quickly. Interviewers were not qualified to assess whether participants could have wheeled independently if given more suitable wheelchairs, skills training or both. Some environmental barriers could be overcome with training. For example, one Kenyan woman said:

*When a person is being given the chair, they need to be trained. We have the first timers, those who do not know anything. It is like taking a child to school and giving him a book and you do not give him a pen. What will he write? At times, you may find some [wheelchair users] stuck on the road simply because they are not aware of what to do, but if they were trained they would have known what to do. [K20]*

Participants added that organizations should provide training to family members and that training should be provided on wheelchair maintenance. A Filipino woman said only her most recently acquired wheelchair had been provided with training related to maintenance:

*Before, I didn’t even have an idea that you can use cooking oil for cleaning the wheelchair and that you should only wipe to clean it and not wash it. That’s the reason why my second wheelchair got rusty. . . I washed and even soaped it. Then, when I got this wheelchair and the instructional book, I learned the proper maintenance, but it was too late [for the previous wheelchair]. [P7]*

However, many participants described receiving a basic toolkit along with the wheelchairs, and some received training in what one Kenyan man called “first aid” for the wheelchair. Maintenance needs also depended on the users’ health, with some reporting performing basic maintenance, while limited hand mobility or other factors made this impossible for others.

### Step 8: Follow-up, maintenance, and repairs

Participants rarely described receiving follow-up services from health care providers or organizations that provided wheelchairs, but they often participated in organizations that supported wheelchair users. Many respondents had difficulty finding a person capable of repairing their wheelchairs, particularly in Kenya, and paying for repairs could be challenging. Sometimes replacement parts were difficult to obtain. Some chairs seemed poorly made and non-functional. Tires were a source of frustration, and users expressed preference for either inflatable or solid rubber tires. Like the receipt of wheelchairs, access to repairs was haphazard. For example, one Filipino man said that the proprietor of a local welding and vulcanizing shop “took pity” and repaired wheelchairs only for the cost of supplies.

On the other hand, for one Kenyan woman, lack of spare parts and maintenance, coupled with wear and tear resulting from wheelchairs not being suited to the environment, resulted in a cycle of obtaining new wheelchairs:

*In that area, we didn’t have people specialized in repairing those wheelchairs, and there were no spare parts. And the environment was full of thorns and that wheelchair had a tube, and it used to get punctures all the time. And so in the process the rim would be damaged, and once it’s damaged and they continue pushing you using that same wheelchair. So what used to happen was the wires would cut, then the tire comes out and the tire falls off. So it was such a challenge I nearly gave up and just decided to stay home. [K10]*

### Other contextual factors

Participants described other services and needs not fitting easily within the WHO service-delivery steps – physical access issues, the need for multiple wheelchairs, the challenges of stigma and the value of peer support.

#### Physical environment as a barrier

The physical environment was often a barrier to wheelchair use. After visiting South Africa, one Kenyan woman contrasted her neighborhood, where public transportation was inaccessible to wheelchair users, with buses in South Africa which are equipped to be wheelchair accessible. Participants talked about needing to request strangers’ help to gain access to public buildings, or carry them upstairs because light rail train stations were inaccessible. In Kenya, participants felt obligated to pay these strangers at times. In both countries, a few participants had modified motorbikes acquired on their own that aided their independent mobility and social and work participation.

#### Multiple chairs for improved access

Study participants frequently had multiple wheelchairs to fulfill different functions and overcome barriers to access. For example, they might have wheelchairs for indoor and outdoor use, or an older wheelchair that they used while showering to prevent a newer wheelchair from rusting. In Kenya, some study participants described needing to keep wheelchairs at their rural homes, because it would not be feasible to transport their wheelchairs with them from the city to a rural area. Sometimes multiple wheelchairs were required because of limitations in wheelchair functionality. For example, study participants might be reluctant to use a wheelchair that was too heavy for them to propel easily alone or one that tipped over easily, but they might keep it in case it was needed. One man from Kenya said, *“This one you can see the wheels wear out, and maybe at that time I don’t have money to replace them or have them fixed, so I will use another one before I get money to fix the other one.”* [K2] In the Philippines, a few study participants described building skateboard-like devices to move about more easily inside their homes. In addition, some participants had acquired wheelchairs for specific activities, like racing or basketball.

#### Stigma

Although the interview guide did not specifically ask about stigma, participants mentioned stigma, particularly those who started using wheelchairs as adults. These users reported that people stared at them in public. For example, a Kenyan woman said, “*I hated going outside because you would find people staring at you and some offering you money like you are a beggar without knowing whether you need it or not or even talking to [you].” [K15]* One woman from the Philippines with mobility recently limited by stroke cried during the interview and expressed worry about being a burden on her family. She said, *“I’m a bother also. I feel ashamed also. Somehow, they must also be getting tired of me. I also seem to be feeling pity for myself. It’s difficult to be like this. More so … it’s better that I died than be like this. I can no longer do everything. It’s like they’re already annoyed with me. Of course, at the very least I’m a bother.”* [P13] However, a man from the Philippines said he experience *less* stigma after moving from a rural province to the Manila metro area. He said, *“Definitely it changed a lot. I tell you, even if we go out, even if I go to [place name] mall, no one notices us. It seems like we’re normal. Unlike in my province that people are like that especially at the mall, at the cinema [staring] like that.”* [P24].

#### Value of peer support

In the absence of follow-up services and formal training in wheelchair skills or maintenance, a key strategy for learning new skills was interacting with other wheelchair users. Peer support from other wheelchair users emerged as an important source of companionship, emotional support, and skill acquisition related to wheelchair use, as well as employment or income generation; this occurred through formal groups for some, and informally for others. A woman from Kenya said, “*The other thing is just the everyday maintenance things, like oiling. I didn’t use to know about that until my wheelchairs joints had rust until they get stuck, because when you wash water goes in. I didn’t know about that until someone asked me, ‘Do you ever oil your wheelchair?’ And I went like, ‘Oh. Am I supposed to?’ This was somebody who was also using a wheelchair and knew that it was important. I do not know where they learnt that from.*” [K12].

This influence was particularly strong in the Philippines since some participants had lived and worked with persons with disabilities. One man from the Philippines said that before moving to Manila and living with other wheelchair users:

“*I had no idea about the correct specifications of a wheelchair. All I care is that I’m using one. And then when I got here, I learned the right specifications of a good wheelchair that I can use. It’s also because of the people I met here. They would advise me, ‘[Name] that’s too wide, have it customized at Metal Craft.’ … Until it came to the point that I became an expert on how I want my wheelchair to be.*” [P21].

## Discussion

### Summary of findings

This study described the experiences of a group of peri-urban and urban wheelchair users in Kenya and the Philippines, to understand how their experiences receiving wheelchair services compared with WHO guidelines. This study aimed to sort people into binary categories of those who had received wheelchairs with services and those who received wheelchairs without services. However, we found that services exist on a wide continuum. Exposure to services may take place over decades and exert a cumulative influence. Personal experience, acquired over years of wheelchair use, also seemed to be an important influence outside receipt of services from others. Another important influence was services received from those outside the formal rehabilitation field, such as peers. Repair services were generally provided by welding and bicycle repair shops. Across diverse backgrounds and medical conditions, most participants described a similar pattern in which their first wheelchairs were unsatisfactory. Later, through exposure to different wheelchairs and perseverance, they learned to seek out wheelchairs that met their needs. This was consistent with the quantitative survey component of this study that found that specific elements of service delivery were associated with improved functioning in both countries [17].

### Study strengths and limitations

Understanding users’ perspectives is an essential aspect of the provision of wheelchairs or any other technology. However, wheelchair provision has tended to rely more on anecdote than research data [4]. This study addresses this crucial gap in the disability research literature.

A limitation of this study is that it asked participants to remember past events, which could lead to recall bias. Also, only one in-depth interview was conducted per respondent, whereas multiple interviews could have improved rapport and led to more detailed responses. Lastly, our sample included only basic wheelchair users, excluding certain types of wheelchairs commonly distributed and some wheelchairs or tricycles specific for longer distance travel. This study only included adults, yet children are an important sector of the wheelchair market, and research into their complex needs and those of their caregivers is scant. More broadly, this study only described wheelchair users’ perception of what services helped their functioning, and the results could yielded more insights into other aspects of wheelchair services and distribution if we had. We did not interview other key informants such as health care providers or wheelchair service providers.

### Findings in context of the published literature

These findings reinforce and expand upon some findings from similar studies. Environmental access, particularly access to public transportation, was a challenge described by other studies [12, 16]. Others have recognized the need for wheelchairs to be adapted to rugged terrains [4, 9] and that currently available wheelchairs often lead to challenges with repair and maintenance [4, 7]. Just as Papadimitriou described the process of “becoming en-wheeled”; participants in this study also described a process of trying to find a wheelchair that became an extension of their bodies [18]. Although the interview guide had minimal emphasis on wheelchair users’ interactions with the public, this subject arose frequently. Like Cahill, we found that wheelchair users experienced stigma at times, and that this was a source of distress, yet they also experienced gestures of kindness and assistance and words of encouragement from strangers [19].

Previous studies in Bangladesh and Indonesia have found that training on how to maneuver wheelchairs was associated with improved outcomes [7, 8]; this is consistent with the survey data from this study which found that training was associated with higher functioning on activities of daily life in the Kenya sample [13, 15]. Wheelchair skills training has also been shown to be beneficial in North America and Europe [20, 21–23]. Informal peer-to-peer instruction in wheelchair use occurred frequently and was perceived to be beneficial. In Canada, Best et al. conducted a pilot study of a formal peer role in training in wheelchair skills and found that the training led to improvements in self-efficacy, wheelchair skills capacity and performance [19]. More broadly, increased social capital has often been linked to improved health outcomes [22]. Improving peer networks could also enable service providers to do outreach to this community more effectively, as their networks could be used to share information and resources. The lack of health professionals available to provide wheelchair services in low- and middle-income countries may be another argument for a peer role in training [23]. In the study settings, self-taught people often are informally teaching other people, leading to a risk of suboptimal practices being shared. Formal peer training models may be a more effective approach; the nonprofit organization Motivation has developed one such training model. Ideally, peers working with trained health-care professionals may provide the most effective model of care.

Borg found that cost was a primary barrier to use of assistive devices [8]. Although most wheelchairs were distributed for free in our study sites, these data suggest that wheelchair users often had to accept unsuitable wheelchairs because they were unable to pay for better quality wheelchairs. However, many only identified them as unsuitable in retrospect, especially when receiving their first wheelchair. In India, Mukherjee found that more than half of donated wheelchairs were discarded and that poorly fitted wheelchairs resulted in pain and injuries [9]. In this study, participants rarely reported discarding wheelchairs, if they were functional, at least until they could obtain a suitable replacement. As in the Mukherjee study, receipt of poorly fitted wheelchairs and injuries related to wheelchair use were commonly described. In Zimbabwe, Visagie et al. observed that adult recipients of donated wheelchair were dissatisfied with their chairs even if they found that the wheelchairs helped them perform daily activities [11]. Similarly, survey data from this study also found that having the fit assessed while the user propelled the wheelchair was associated with higher performance of activities of daily living, compared with those who did not receive this service [13, 15].

Previous research has suggested that a shortage of wheelchairs leads many to go without wheelchairs. One three-country study found that, prior to receiving their current wheelchairs, 90% of recipients had spent most time sitting in chairs or lying in beds [24]. By including participants who had received their current wheelchair six months to five years ago, we expected to sample many participants who had spent extended periods of time in need of wheelchairs but were unable to obtain them. In fact, this scenario was rarely described. Many respondents had multiple wheelchairs that they used for different purposes, such as moving around within the house or outside the house. This discrepancy may be because all of our respondents lived in major urban centers in countries that benefit from international donors’ contributions and this enabled them to have better access to wheelchairs and accompanying services. It is also possible that disadvantaged populations existed within these settings and that our sampling strategies did not reach them.

### Recommendations for future research and programs

Although this study has in many ways focused on the challenges of wheelchair provision in these settings, participants acknowledged that they received wheelchairs that would have been difficult or impossible for them to pay for and, despite challenges, they were grateful to have some means of mobility. This study highlights some key information for the research community and needs for future research, among an underserved population where systematic descriptions of available services have been scarce. Future qualitative studies should seek to include caregivers and service providers in addition to wheelchair users, as their perspectives are essential to the success of wheelchair service programs. Recruitment was challenging because wheelchair service organizations have limited capacity for recordkeeping, particularly in Kenya. Creating a registry of wheelchair users and updating that registry consistently could facilitate both research and follow-up services. Respondents suggested that disabled persons organizations or other organizations create a searchable network or database of service providers, repair shops, and other resources; this information could also identify underserved areas and then try to increase services in those areas.

### Implications for provision of mobility devices and other types of technology

These findings have implications not just for mobility devices but for other new technologies that are introduced into less resourced settings. Whenever a product is introduced to a new market, it is essential to understand the user population. Obtaining users’ perspectives is critical to ensuring adequate access. The approach used in this study may also be applicable to other new technologies, since the concerns they face are the same for any innovation that has failed to achieve scale. Although the service steps have been outlined, and donors are available to provide wheelchairs questions remain about who will provide the services, how to train or license providers, and where they will work. It will also be important to consider to what extent technology could play a part in expanding service reach, whether task sharing and whether a need exists to increase demand. Current service provision models should be examined to identify who can access the services, who is currently left out, and how inclusion can be increased. Future studies may include a review of product safety standards, the range of available products and what models, incentives, and training are needed to support the maintenance and repair of the product. Further work can be done to fully understand the challenges posed by the physical environment and the potential policy implications.

## Conclusions

This qualitative study allowed wheelchair users to describe their experiences in their own words and to contribute their views on what services would be valuable for themselves and other wheelchair users. Given the limited resources in low and middle income countries, it will be crucial to establish efficient models for service delivery. In addition to providing wheelchairs and services to individual wheelchair users and their families, intervention must also be directed toward environmental factors, such as barriers in the physical environment and perceived social stigma, and toward fostering peer support.

## Additional file

Additional file 1: In-Depth Interview Guide. (DOCX 24 kb)
