# Supplementary material for: Perspectives of basic wheelchair users on improving their access to wheelchair services in Kenya and Philippines: a qualitative study
Source: BMC Int Health Hum Rights. 2017 Aug 17;17:22. doi: 10.1186/s12914-017-0130-6 (PMC5561585; doi:10.1186/s12914-017-0130-6)
Supplement: Additional file 1: — In-Depth Interview Guide. (DOCX 24 kb) [file 12914_2017_130_MOESM1_ESM.docx]

**In-Depth Interview Guide: Cover Sheet**

**Date:**

Participant Study ID: ___________ (3-letter org prefix and number id)

*(Circle one in each row)*

Gender: M F

Service or Distribution: S D

Age: ≤45 >45

*ASK AT THE END OF INTERVIEW:*

Highest educational level:

*Notes to interviewer:*

- *It is not necessary to ask every single prompt. Choose from among the prompts if the information is not forthcoming.*
- *The order of the interview can be modified, according to topics raised by the participant. You can go back to a previous topic or go to a future topic, follow the conversation.*
- *Please tell the intereviewee that he or she is the expert, we will learn from him or her, and we would like to hear their experiences; there are no right or wrong answers.*
- *Clearly explain the study purpose to the participant and his or her caregiver (if present) and obtain consent before starting interview; ask for permission to interview in private, if at all possible.*
- *Write notes after the interview on the interview environment (for example, interruptions, others being present, disturbances, any issues that arose). Do the transcript from the audio recording as soon as possible.*

**Qualitative Field Guide Questions for Wheelchair Users**

1. Please tell me about the **circumstances or condition** that required you to need a wheelchair.

- Age when the loss of mobility occurred.

1. Tell me about your **first** wheelchair.

*Prompts*:

- Describe how you got the wheelchair: Organization, services that provided it, cost, etc.
- Describe the wheelchair and cushion.
- Describe the process used to select the chair.
- Describe any services or training received to help use the chair.
- Describe any services or training received to help take care of the chair.
- Describe how you used the chair.
- Describe any challenges and successes in the chair.

1. How has your **life changed** since you first received a wheelchair?

*Prompts*

- What goals have you reached, and what remain?
- What has helped you reach your goals, and what could help you further?
- What kind of involvement have you had with other persons with disabilities? (For example, membership in a disabled persons organization.)
- What kind of contacts have you had with organizations that help wheelchair users, other than the time that you received the wheelchair?

1. How many **total wheelchairs** have you owned? (*if the person has owned more than one wheelchair*.)
2. Since you received your first wheelchair up to now, what have been your memorable experiences (best or worst experiences) with…
   1. The wheelchair service provider?
   2. … family caregivers?
   3. … community support? (friends, neighbors, community members etc.)
   4. …physical environment in your home or around your home?
3. Tell me about your **current** wheelchair (*if the person has owned more than one wheelchair*.)

*Prompts*:

- What led you to change from the previous wheelchair?
- Describe how you got the wheelchair: Organization, services that provided it, cost, etc.
- Describe the wheelchair and cushion.
- Describe the process used to select the chair.
- Describe any services or training received to help use the chair.
- Describe any services or training received to help take care of the chair.
- Describe how you used the chair.
- Describe any challenges and successes in the chair.

1. What is a **typical day** in your life like now?
2. Tell me about your **favorite** wheelchair (*if the person has owned more than one wheelchair*.)

*Prompts*:

- How was (is) this chair different from other chairs?
  - Did the provider of this chair do anything that made a difference?
  - Tell me about a time this chair helped you reach a goal. (How does this chair help you meet your daily goals?)

1. Tell me about your **least favorite** wheelchair (*if the person has owned more than one wheelchair*.).

*Prompts*:

- How was (is) this chair different from other chairs?
- Did the provider of this chair do anything that made a difference?
- Tell me about a time this chair made it hard for you to reach a goal. (How does this chair help you meet your daily goals?)

1. What do you recommend for **wheelchair services**– special help that a wheelchair user receives along with a wheelchair?

*Prompts*

- - Are needs different after a wheelchair user receives their second or third wheelchair?
  - What services or training should a wheelchair user receive?
  - What services or training should a family (caregiver) receive?
  - How would you describe an ideal wheelchair service provider. Why do you say that?
